# Building Minimized Epigenetic Clock by iPlex MassARRAY Platform

**DOI:** 10.3390/genes15040425

**Published:** 2024-03-28

**Authors:** Ekaterina Davydova, Alexey Perenkov, Maria Vedunova

**Affiliations:** Institute of Biology and Biomedicine, Lobachevsky State University, 23 Gagarin Ave., Nizhny Novgorod 603022, Russiamvedunova@yandex.ru (M.V.)

**Keywords:** aging, CpGs, DNA methylation, epigenetic clocks, MassARRAY

## Abstract

Epigenetic clocks are valuable tools for estimating both chronological and biological age by assessing DNA methylation levels at specific CpG dinucleotides. While conventional epigenetic clocks rely on genome-wide methylation data, targeted approaches offer a more efficient alternative. In this study, we explored the feasibility of constructing a minimized epigenetic clock utilizing data acquired through the iPlex MassARRAY technology. The study enrolled a cohort of relatively healthy individuals, and their methylation levels of eight specific CpG dinucleotides in genes *SLC12A5*, *LDB2*, *FIGN*, *ACSS3*, *FHL2*, and *EPHX3* were evaluated using the iPlex MassARRAY system and the Illumina EPIC array. The methylation level of five studied CpG sites demonstrated significant correlations with chronological age and an acceptable convergence of data obtained by the iPlex MassARRAY and Illumina EPIC array. At the same time, the methylation level of three CpG sites showed a weak relationship with age and exhibited a low concordance between the data obtained from the two technologies. The construction of the epigenetic clock involved the utilization of different machine-learning models, including linear models, deep neural networks (DNN), and gradient-boosted decision trees (GBDT). The results obtained from these models were compared with each other and with the outcomes generated by other well-established epigenetic clocks. In our study, the TabNet architecture (deep tabular data learning architecture) exhibited the best performance (best MAE = 5.99). Although our minimized epigenetic clock yielded slightly higher age prediction errors compared to other epigenetic clocks, it still represents a viable alternative to the genome-wide epigenotyping array.

## 1. Introduction

DNA methylation is based on the transfer of a methyl group to position C-5 of the cytosine ring with the formation of 5-methylcytosine (5mC). DNA methylation primarily occurs at CpG sites (CpGs) and follows a symmetrical pattern. However, non-CpG methylation when other nucleotides are present adjacent to 5mC instead of guanine can also occur [1].

The interplay between DNA methylation and other epigenetic mechanisms, such as histone modifications and non-coding RNA, collectively regulates genome function without altering the underlying DNA sequence. Disruptions in gene expression patterns controlled by these epigenetic mechanisms have been implicated in various diseases, including autoimmune disorders, cancer, and premature aging [2].

**Figure 1:**
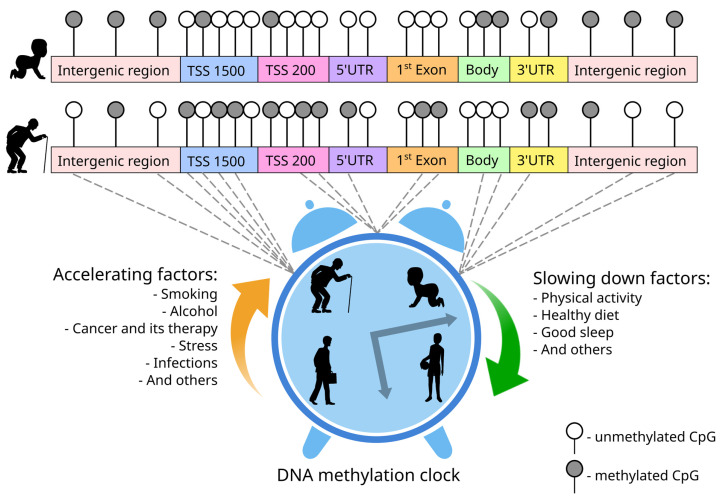
Schematic representation of the DNA methylation clock. During aging, methylation level of some CpGs changes non-stochastically. Some CpGs demonstrate a rise of methylation level (hypermethylated CpGs), while others show a decrease (hypomethylated CpGs). Such CpGs can be located in different parts of genes and intergenic regions; however, for instance, hypermethylated CpGs are often associated with TSS and hypomethylated ones with body. Complex of age-associated hypermethylated and hypomethylated CpGs forms the basis of the methylation clock, the course of which is influenced by many factors.

Specific epigenetic changes are known to be one of the key features of aging [3]. The predominant trend of age-related changes in DNA methylation is the hypermethylation of CpG-rich promoter sequences and the hypomethylation of genes with a low content of CpGs [4]. Studies on identical twins have demonstrated that, as they age, their DNA methylation profiles become increasingly divergent. This phenomenon, known as epigenetic drift, can be influenced by environmental factors, as well as disruptions in the activity of enzymes involved in the methylation process [5,6]. Epigenetic drift leads to unpredictable changes in DNA methylation patterns among aging individuals. However, some changes appear to be systematic and may or may not occur in a tissue-specific manner [7]. This suggests that some of the age-related changes in DNA methylation are not stochastic, but are associated with certain biological mechanisms [8]. Thus, the methylation levels of specific CpGs exhibit a strong correlation with age. Collectively, the methylation status of these sites can be considered an “epigenetic clock” that provides insights into both chronological and biological age (Figure 1).

The conventional approach for building epigenetic clocks involves using genome-wide DNA methylation analysis, which is rather redundant since it assesses the methylation levels at a large number of CpGs (over 850,000 CpGs). The cost of these assays is a major limitation of their application. As a result, there is growing interest in developing more cost-effective approaches that utilize a smaller number of CpGs [9,10].

### Minimized Epigenetic Clock

The use of genome-wide methylation analysis on methyl chips (Illumina, San Diego, CA, USA) has gained popularity in the development of epigenetic clocks. These clocks include the Bocklandt clock [11], which assesses DNA methylation in saliva, the Horvath multi-tissue clock [12], the Hannum clock for whole blood [13], the Zhang clock for whole blood and saliva [14], the Boroni Skin clock for skin [15], and others. Most of these epigenetic clocks utilize a large number of CpGs, which enhances the accuracy of age prediction. However, the cost and complexity associated with this technology limit its widespread application. To address these limitations, more cost-effective approaches have been developed, focusing on the assessment of the methylation levels at a smaller number of CpGs that show the strongest associations with age. These “minimized” clocks assess the level of methylation using bisulfite pyrosequencing, quantitative PCR, EpiTYPER technology (Agena Bioscience, San Diego, CA, USA), SNaPShot, and others [9]. Table 1 provides a comparison of commonly used technologies for building minimized epigenetic clocks.

On the basis of pyrosequencing, the blood aging clock was created by assessing the methylation levels of only three CpGs located in the *ITGA2B*, *ASPA*, and *PDE4C* genes [16]. Similarly, the Bekaert, Thong, Garali MQR, and Garali GBR clocks are based on the pyrosequencing of 2–4 CpGs in the promoters of the *ASPA*, *EDARADD*, *ELOVL2*, *KLF14*, *PDE4C*, and *TRIM59* genes [10]. Pyrosequencing is a method that relies on bisulfite conversion, which allows us to artificially create single-nucleotide polymorphisms (SNPs) at the sites of CpGs. After bisulfite conversion and amplification, the DNA sequence is subjected to sequencing. During this process, DNA polymerase incorporates deoxynucleotide triphosphates (dNTPs) into the growing chain, releasing pyrophosphate, which is then converted to ATP by ATP sulfurylase. Subsequently, ATP triggers an enzymatic reaction that results in the emission of a quantum of light. The degree of methylation is determined by comparing the light emission peaks when a C or T is incorporated at the CpG site region [17].

There are also minimized clocks that allow us to estimate the age of multiple tissues using the SNaPShot method. This technology was utilized to determine the DNA methylation levels of five CpGs in the *ELOVL2*, *FHL2*, *KLF14*, *C1orf132/MIR29B2C*, and *TRIM59* genes in blood, saliva, and buccal epithelium samples [18]. The authors observed a strong correlation between the predicted age and chronological age not only within each tissue type, but also in the combined model (r = 0.937). The SNaPShot technology involves a bisulfite conversion step, followed by the amplification of the target fragments and a single-nucleotide extension reaction. The products of the single-nucleotide extension reaction are then analyzed using capillary electrophoresis [19].

The quantitative PCR method for assessing the methylation method (MS-qPCR), which utilizes the bisulfite processing of DNA, is another approach for assessing methylation. Quantitative PCR based on fluorescence is used to calculate the level of methylation. This method is suitable for analyzing the methylation level of DNA fragments that may contain multiple CpGs [20]. The methylation values obtained by bisulfite pyrosequencing and MS-qPCR were compared [21]. The results demonstrated that the degree of methylation measured by MS-qPCR was lower for methylation levels ranging between 0–15% and higher for values over 30% compared to pyrosequencing.

The biological age estimation model based on the methylation patterns of seven groups of adjacent CpGs using the EpiTYPER method has been proposed [22]. This model showed a high correlation with chronological age (r = 0.89), as well as the rate of aging (accelerated/decelerated). Based on this technology, an expanded age prediction model spanning from early childhood to longevity was proposed [23]. The EpiTYPER technology involves amplifying bisulfite-treated DNA, transcribing it into RNA, and fragmenting it into different-molecular-weight fragments depending on the methylation state. The resulting fragments are then analyzed using matrix-assisted laser desorption/ionization time-of-flight mass spectrometry (MALDI-TOF MS) [24].

The EpiTYPER technology shares many similarities with the iPlex assay (MassARRAY system), also based on MALDI-TOF MS. The iPlex assay employs a single-nucleotide primer extension approach, where the extension of a primer to a specific dideoxynucleotide triphosphate (ddNTP) depends on the methylation status of a particular CpG site [25]. Although there are similarities between the EpiTYPER and iPlex assay (MassARRAY system) technologies, we were unable to find information on the development of an epigenetic clock specifically based on the iPlex assay method. However, the iPlex assay is widely used for SNP genotyping and the DNA methylation assessment in various studies [26,27,28,29]. A good correlation was reported between methylation values obtained by the iPlex assay and results obtained from the Illumina 450K array and the EpiTYPER method [30]. However, sometimes, the authors could not obtain reproducible results with the iPlex assay, while the MiSeq reproducibility was acceptable (standard deviation ranging from 1.1% to 2.42% for six replicates) [31].

The choice between EpiTYPER and iPlex assay technologies depends on the specific goals of the study. When aiming to analyze multiple CpGs within a single amplicon, the EpiTYPER technology is more cost-effective. On the other hand, if the study intends to evaluate numerous CpGs dispersed throughout the genome, the iPlex assay technology is more suitable [24]. In our study, as we analyzed CpGs located in various genes and intergenic regions, we opted to use the iPlex assay technology.

A common feature of the described technologies is their dependence on the bisulfite conversion of DNA. This method is based on the conversion of unmethylated cytosine to uracil, while methylated cytosines in the CpGs remain unchanged [32]. Initially, the sulfonation of unmethylated cytosine residues occurs, followed by deamination, resulting in the formation of uracil sulfonate. Subsequently, the desulfonation of uracil sulfonate takes place, resulting in the formation of uracil [33]. Despite the widespread use of the bisulfite conversion method and its significance in various DNA methylation assessment technologies, it has some significant limitations. The method requires high DNA concentrations, as exposure to harsh conditions leads to DNA degradation [34,35]. Additionally, the complete conversion of unmethylated cytosines is crucial, as incomplete conversion may result in biased results [36].

The development of a universal model for age prediction that can be applied regardless of the specific technology used to determine DNA methylation is currently being explored. EpiTYPER, SNaPShot, pyrosequencing, and MiSeq technologies were compared [35]. The results indicated a high level of comparability between the EpiTYPER, pyrosequencing, and MiSeq systems. However, the SNaPShot technology demonstrated larger differences in the obtained results.

In the present study, we aim to investigate the feasibility of constructing a minimized epigenetic clock using data from single CpGs obtained through iPlex MassARRAY technology. Additionally, we assess the performance of this clock by analyzing its compatibility with Illumina 450K and EPIC genome-wide data.

**Table 1:** Comparison of some technologies for assessing DNA methylation in “minimized” clocks.

References	Disadvantages	Advantages	Technology
[20,35,37]	Short amplicons (150–200 bp) Dedicated and expensive equipment Problems with high-density CpG Difficult to analyze multiple markers at the same time	Highly quantitative Single-site resolution Fast run times Detects differences in methylation with an accuracy of up to 0.5%	Pyrosequencing
[20,35]	Semi-quantitative technology Possible bias of detected methylation values due to different ddNTP fluorescence intensity	High throughput Rapid quantitation of cytosine methylation Multiplexing capability	SNaPShot
[20,23,24]	Large amounts of genomic DNA (300 ng) The average percentage of methylation is determined if the studied points are located close to each other Possible influence of SNP on the degree of DNA methylation Dedicated equipment Sequence fragmentation may exclude some CpGs	Reproducible Fast run times Determines differences in methylation with an accuracy of 5–7% Allows simultaneous analysis of multiple CpGs in a specific area	EpiTYPER (MassARRAY system)
[20,36]	Low precision No single-site resolution Difficulties with the selection of primers and conditions	Simplicity Sensitive Quantitative and qualitative Equipment is easily accessible Can be multiplexed	Quantitative PCR (MS-qPCR)
[20,24,26]	Dedicated equipment Lack of automatic processing of results The need for optimization to improve the accuracy of genotyping	Multiplexing capability (analysis of multiple CpGs in different regions) Small amount of test sample Low launch costs Determines differences in methylation with an accuracy of 5–7%	iPlex assay (MassARRAY system)

## 2. Material and Methods

### 2.1. Materials

The study utilized a total of 131 peripheral blood samples obtained from relatively healthy volunteers in Nizhny Novgorod, Russia. The samples were collected using K3-EDTA as an anticoagulant. In our research, we formed two datasets. The first dataset is UNN EPIC (n = 131) and the DNA methylation level in this dataset was performed using the Illumina EPIC array method. The second one is the UNN MassARRAY dataset (n = 50), where DNA methylation level was performed using the iPlex MassARRAY method. Moreover, the MassARRAY dataset is a subset of the UNN EPIC dataset (both methods were used in order to establish DNA methylation of 50 people).

The age range of the subjects in the UNN EPIC dataset was from 15 to 101, while, in the UNN MassARRAY dataset, it was between 25 and 84 years. The proportion of women in the UNN EPIC dataset was 42%, and, in the UNN MassARRAY dataset, it was 58%.

The UNN MassARRAY dataset served as training data, and the UNN EPIC dataset as test data, as well as open datasets on whole blood methylation in a large number of healthy people of different ages: Illumina 450K (GSE87571, GSE40279, and GSE55763) and Illumina EPIC (GSE152026).

### 2.2. CpGs Selection

Eight CpGs were selected for analysis that are included in all popular epigenetic clocks and have a strong correlation with age. These CpGs included cg07547549 (*SLC12A5*), cg08262002 (*LDB2*), cg01620164 (*FIGN*), cg11649376 (*ACSS3*), cg16008966 (intergenic), cg06639320 (*FHL2*), cg14556683 (*EPHX3*), and cg22454769 (*FHL2*). The selection of CpGs was based on the analysis of healthy human methylation datasets GSE87571, GSE40279, GSE55763, and GSE152026.

### 2.3. Primer Design for iPlex MassARRAY

The flanking sequences for each selected CpG dinucleotide were obtained using the UCSC genomic browser based on the GRCh37/hg19 assembly of the human genome. A region of interest consisting of 100 base pairs (bp) upstream and downstream of the target CpG dinucleotide was chosen. The design of PCR primers and extension primers (Table S1) was performed using the Assay Design Suite v2.0 software (Agena Bioscience, San Diego, CA, USA). Prior to importing the sequences into the Assay Design Suite, the CpGs of interest were designated as SNPs ([C/T]G). Any SNPs identified by the Assay Design Suite were labeled according to the IUPAC nomenclature. CpGs that were not of interest were designated as NG, and all cytosines were replaced with thymines to simulate bisulfite conversion.

It should be noted that, if SNPs were present in the annealing region of the extension primers, primers containing inosine (I) were utilized since they are capable of effectively binding to the target genomic region regardless of the allele at the SNP site.

### 2.4. DNA Methylation Analysis of CpGs by iPlex MassARRAY

Genomic DNA was extracted from peripheral blood cells using the GeneJET kit following the manufacturer’s protocol (Thermo Fisher Scientific, Waltham, MA, USA). The concentration and purity of the isolated DNA were determined using a Qubit bench fluorimeter (Thermo Fisher Scientific, Waltham, MA, USA) and a NanoDrop One spectrophotometer (Thermo Fisher Scientific, Wilmington, DE, USA). The isolated DNA was then subjected to bisulfite conversion using the EZ DNA Methylation Kit (Zymo Research, Tustin, CA, USA), following the manufacturer’s instructions. Briefly, 500 ng of genomic DNA was treated with bisulfite, and the modified DNA was eluted with 30 μL of water. Methylation analysis was performed, employing MALDI-TOF-MS technol-ogy with the MassARRAY system (iPLEX assay, Agena Bioscience, San Diego, CA, USA) according to the manufacturer’s protocol. The obtained results were analyzed using the MassARRAY Typer Analyzer 4.0 software provided (Agena Bioscience, San Diego, CA, USA.

The size and quality of products after the first PCR were visualized on 1.5% agarose gels with ethidium bromide under ultraviolet (UV) light.

The method for methylation assessment is based on the detection of products generated after the primer extension reaction. Initially, locus-specific PCR is carried out using a pair of primers designed for the region of interest. Subsequently, a second PCR is performed using ddNTPs. Moreover, there is shrimp alkaline phosphatase (SAP) step between the first PCR and second one. SAP catalyzes the removal of phosphate groups from the 5′ ends of dNTPs that enhance the single-nucleotide primer extension reaction [38].

**Figure 2:**
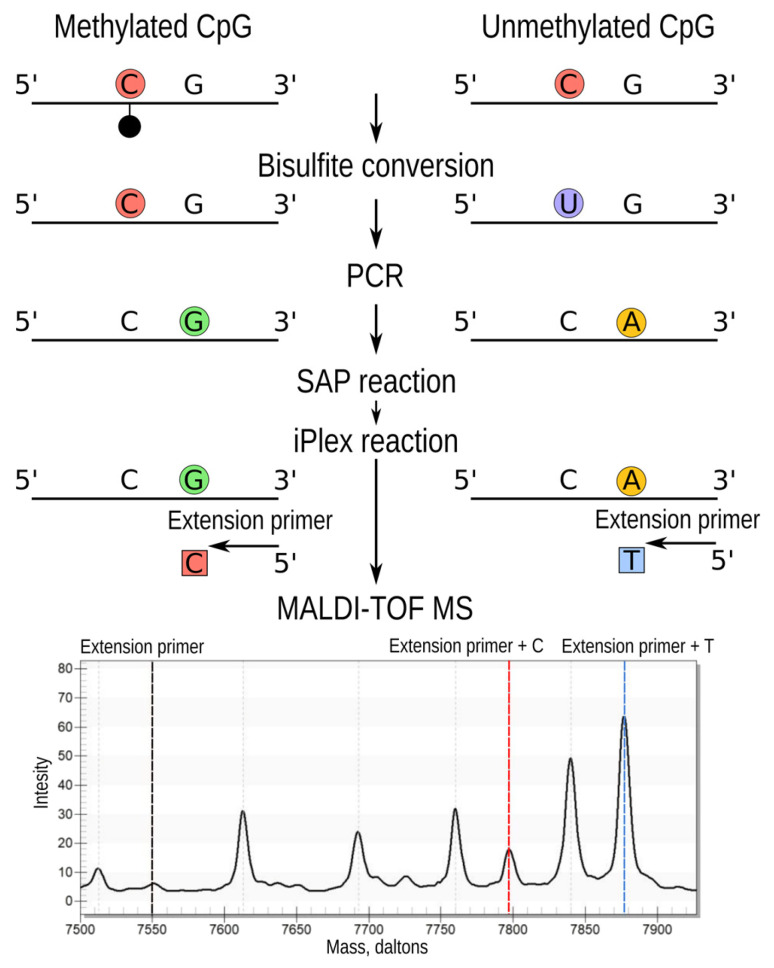
The scheme for determining methylation by MALDI-TOF MS. At the initial stage, DNA is isolated from blood cells. The next step is bisulfite conversion, which makes it possible to distinguish between methylated and unmethylated cytosine. The unmethylated cytosine is converted to uracil, while the methylated one is not changed. During PCR, the methylated CpGs are converted to guanine and the unmethylated ones are converted to adenine. The SAP reaction step is needed to prevent embedding of remaining dNTP during the iPlex reaction. On the iPlex reaction step, the extension primer elongates by one terminating nucleotide, and then the resulting fragments having different masses are analyzed on a mass spectrometer.

During the second PCR, the extension primer is annealed near the CpG site of interest and is extended by one nucleotide. The elongation products have varying masses depending on the nucleotide incorporated, reflecting the methylation status of the CpG site in the original DNA sample (Figure 2).

The methylation level was determined by calculating the ratio of peaks corresponding to primer extension products. In the case of CpGs being methylated, the primer was extended with cytosine, while, in the unmethylated state, it was extended with thymine. The methylation level was calculated using the formula: A/(A + B) × 100%, where A represents the relative intensity of the cytosine peak, and B represents the relative intensity of the thymine peak.

In our study, the DNA methylation assessment protocol was optimized after an initial assessment of the repeatability of the obtained results. The final protocol was committed only after achieving intraclass correlation coefficient (ICC) greater than 0.9 (calculated in R using the icc() function).

### 2.5. Methylation Assessment by the Illumina EPIC Array Method

Phenol Chloroform DNA extraction was performed on blood samples from the UNN EPIC dataset. DNA was quantified using the DNA Quantitation Kit Qubit dsDNA BR Assay (Thermo Fisher Scientific, Waltham, MA, USA) and 250 ng was bisulfite-treated using the EpiMark Bisulfite Conver-sion Kit (NEB, Ipswich, MA, USA) with case and control samples randomly distributed across arrays. The Illumina Infinium MethylationEPIC BeadChip (Illumina, San Diego, CA, USA) was used according to the manufacturer’s instructions.

### 2.6. Data Processing

Statistical analysis of the obtained results was carried out using R (4.0.4). For statistical processing, nonparametric analysis methods were employed, and the normal distribution of data was assessed prior to analysis. Differences were considered statistically significant at *p* < 0.05. The illustrations were generated using the InkScape (1.0.2) and Matplotlib (3.3.4) software programs.

All DNA methylation datasets were preprocessed using the ChAMP R package version 1.10.0 [39]. First, probes with a detection *p*-value above 0.01 in at least 10% of samples were removed. Second, probes with a beadcount less than three in at least 5% of samples were removed. Third, all non-CpG probes [40], SNP-related probes [41], and multi-hit probes were removed [42]. Fourth, all probes located on chromosomes X and Y were filtered out. All dataset with raw.idat data files available were normalized using functional normalization [43]. The total number of subjects in each dataset, as well as the number of probes remaining after preprocessing, are presented in Table S2.

A minimized epigenetic clock was constructed using iPlex MassARRAY technology by employing models from different classes, including the widespread linear model elastic net [44], gradient-boosted decision tree (GBDT)—extreme gradient boosting (XGBoost) [45], light gradient-boosting machine (LightGBM) [46], categorical boosting (CatBoost) [47] and deep neural network (DNN)—multilayer fully connected networks with various layer architectures, TabNet [48], and feature tokenizer and transformer (FT-Transformer) [49]. Our small iPlex MassARRAY clocks are based on methylation level of eight CpGs (CpG cg08262002, CpG cg11649376, CpG cg16008966, CpG cg06639320, CpG cg22454769, CpG cg07547549, CpG cg01620164, and CpG cg14556683).

## 3. Results

### 3.1. Correlation between DNA Methylation Level of Studied CpGs and Chronological Age

**Figure 3:**
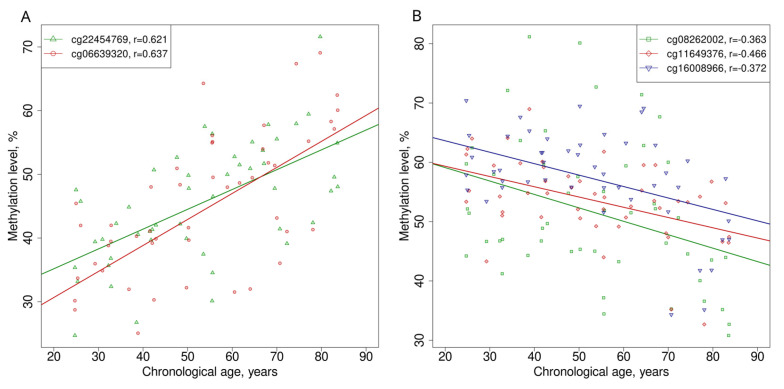
Methylation level of studied CpGs depending on chronological age in UNN MassARRAY dataset: (**A**) hypermethylated CpGs; and (**B**) hypomethylated CpGs. All presented CpGs have significant correlation with chronological age.

In the initial stage of the study, CpGs were selected based on their methylation level’s strong correlation with age in healthy individuals from publicly available methylation datasets (GSE87571, GSE40279, GSE55763, and GSE152026). To investigate the relationship between DNA methylation levels obtained using the iPlex MassARRAY technology and chronological age, a correlation analysis was performed using the Spearman rank correlation method (Figure 3). The results revealed significant correlations between age and the methylation level of the following CpGs: cg08262002 (r = −0.363; *p* < 0.01), cg11649376 (r = −0.466; *p* < 0.001), cg16008966 (r = −0.372; *p* < 0.01), cg06639320 (r = 0.637; *p* < 0.001), and cg22454769 (r = 0.621; *p* < 0.001). However, no significant correlation between chronological age and DNA methylation level was observed for CpGs cg07547549 (r = 0.184), cg01620164 (r = 0.150), and cg14556683 (r = 0.268) (*p* > 0.05).

### 3.2. Comparison of Methylation Results Obtained by iPlex MassARRAY Technology and Illumina EPIC Array

**Figure 4:**
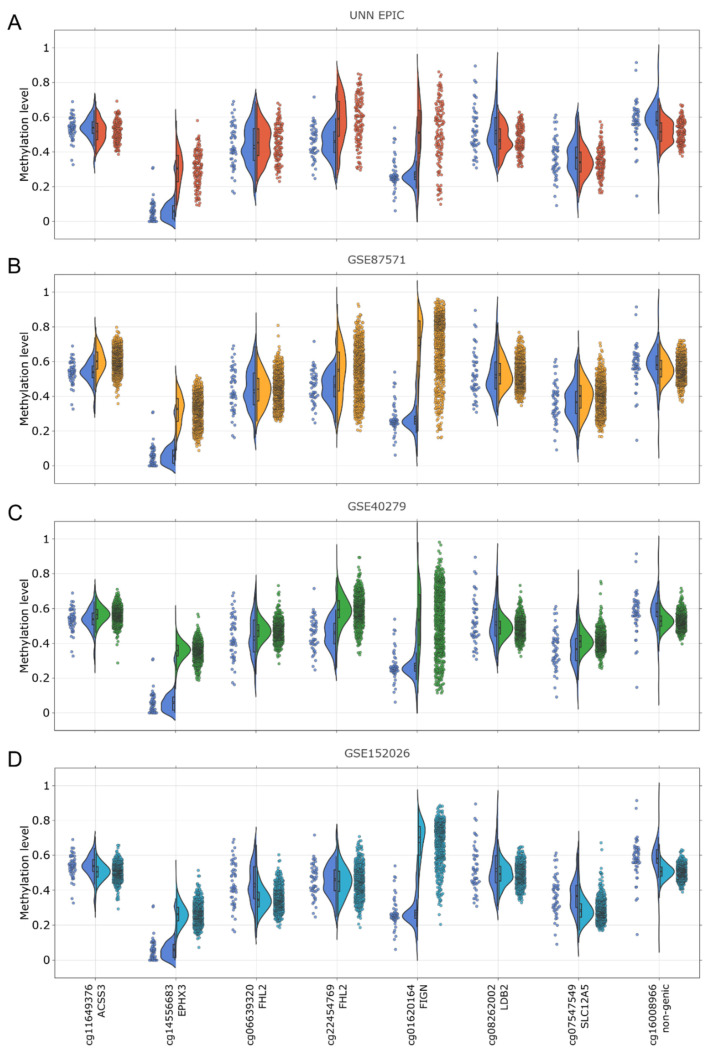
Distributions comparison of DNA methylation level of 8 CpGs from UNN MassARRAY (the left side of violin plots) with values from Illumina datasets (the right side of violin plots): (**A**) UNN MassARRAY (n = 50) vs. UNN EPIC (n = 131); (**B**) UNN MassARRAY vs. GSE87571 (n = 729); (**C**) UNN MassARRAY vs. GSE40279 (n = 656); and (**D**) UNN MassARRAY vs. GSE152026 (n = 519).

Initially, we selected CpGs that had a high correlation between age and methylation level based on Illumina data. However, in our study, we did not observe the same correlation between age and methylation level for some of these CpGs. That is why we compared the methylation levels of eight CpGs from the UNN MassARRAY, UNN EPIC, and Illumina datasets (Figure 4). CpG cg11649376, cg06639320, cg22454769, cg08262002, cg07547549, and cg16008966 exhibited comparable ranges of methylation values across all three datasets. However, the methylation values for CpG cg14556683 and cg01620164 were found to be lower in the UNN MassARRAY dataset compared to both the UNN EPIC and Illumina datasets.

**Figure 5:**
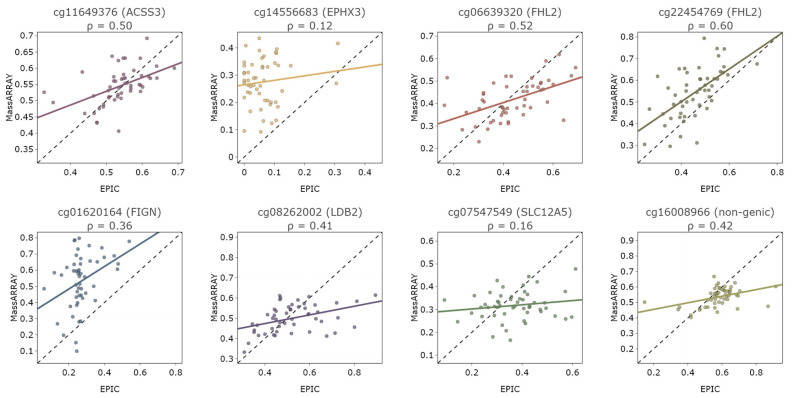
Correlation between methylation values in UNN MassARRAY and UNN EPIC datasets. Every point corresponds to the DNA methylation level of one sample measured by the iPlex MassARRAY technology and Illumina EPIC array.

Additionally, we compared the data obtained using the iPlex MassARRAY technology (UNN MassARRAY dataset) and the Illumina EPIC array (UNN EPIC dataset) (Figure 5). The maximum correlation coefficients observed were 0.5, 0.52, and 0.6 for CpG cg11649376, cg06639320, and cg22454769, respectively. For the methylation level of CpGs that did not exhibit a significant correlation with age (cg07547549, cg01620164, and cg14556683), the relationship between the methylation values in the UNN MassARRAY and UNN EPIC datasets was weak.

### 3.3. Small iPlex MassARRAY Clocks

The training and validation of the models were performed on the UNN MassARRAY dataset. A five-fold cross-validation approach was employed, resulting in the mean absolute error (MAE) being computed for each cross-validation split, along with the standard deviation of this error. The best model on a specific split, determined by the minimum MAE value, was selected. Illumina data from the GSE87571, GSE40279, GSE55763, GSE15026, and UNN EPIC datasets were used as test data. Table 2 presents the results of the regression of chronological age according to the MassARRAY data, where highlighted row corresponds to the best model values (TabNet).

The best GBDT models and the best neural networks showed similar results: the TabNet and FT-Transformer neural network architectures, along with the CatBoost GBDT model, exhibit comparable MAE values, not only on the validation dataset, but on all test datasets. The CatBoost model performed well on the GSE87571 and GSE55763 datasets, but showed relatively weaker performance on the GSE40279 dataset and both Illumina EPIC datasets; it particularly excelled on the GSE15026 dataset. Comparing the neural network models, the FT-Transformer slightly (for less than a year) outperformed TabNet across all test datasets, except for the GSE87571 dataset. However, considering the primary evaluation criterion, which includes the MAE on the validation set and the average error across all cross-validation splits, the TabNet architecture exhibited the best overall performance. Therefore, it was selected as the main model for further analysis.

Next, we compared our MassARRAY Age Clock with other epigenetic models. Four epigenetic age estimators and five of its PC-variations from Horvath’s calculator (DNA Methylation Age Calculator Available online: https://dnamage.genetics.ucla.edu/home/ (accessed on 28 January 2024) were employed in the study: DNAmAgeHannum [15], DNAmAge [11], DNAmPhenoAge [16], and DNAmGrimAge [17]. The DNAmAgeHannum model quantifies the aging rate of human methylome in whole blood. The DNAmAge multi-tissue age predictor provides estimates of DNA methylation in various tissues and cells. DNAmPhenoAge, a biomarker of aging, was developed by considering composite clinical measures of phenotypic age [16]. DNAmGrimAge is a composite biomarker based on DNAm surrogates of seven plasma proteins and smoking history. Figure S1 demonstrates indicators of various epigenetic clocks for the GSE87571, GSE40279, GSE55763, and GSE152026 datasets.

Table 3 presents the results of all epigenetic clock models, with the minimum MAE values highlighted in color. Our minimized clocks based on the TabNet model consistently demonstrate higher MAE values compared to the other epigenetic clocks. Despite this, the MAE values of our minimized clocks are comparable to the MAE of other epigenetic clocks, which indicates the possibility of using our model.

### 3.4. Genomic Localization of Age-Associated CpGs

**Figure 6:**
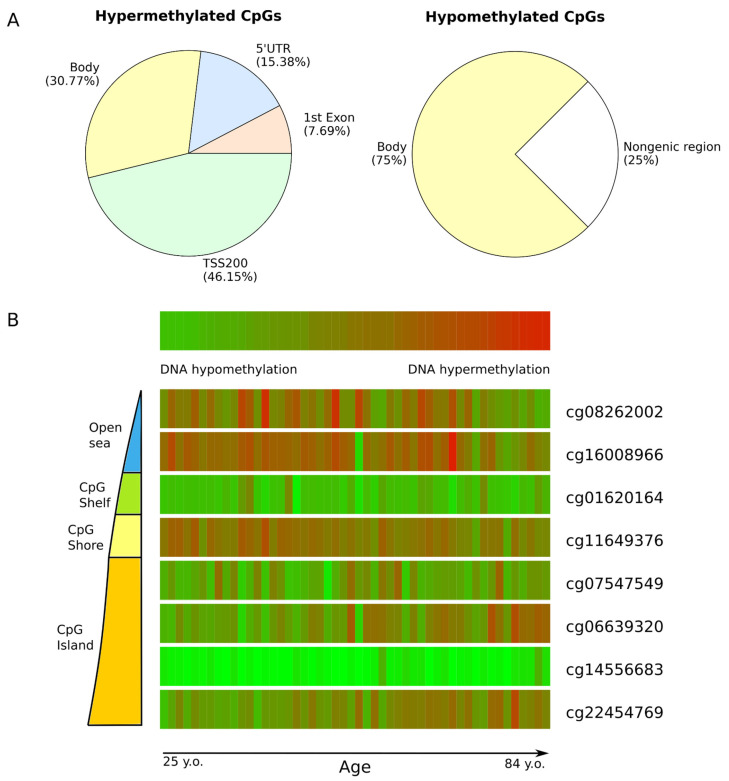
Location of studied DNA methylation sites: (**A**) percentage distribution of studied CpGs in the gene structure; and (**B**) schematic distribution of studied CpGs in relation to the structure of the CpG island in UNN MassARRAY dataset.

Figure 3 shows that some CpGs are characterized by age-dependent hypermethylation (cg22454769 and cg06639320), while others display hypomethylation (cg11649376, cg08262002, and cg16008966). It is assumed that the genomic localization of hyper- and hypomethylation sites is different. The literature has indicated that hypermethylation predominantly occurs within CpG islands, whereas hypomethylation is more frequently observed in CpGs outside of CpG islands [43]. To examine the relationship between hyper- and hypomethylation sites and their genomic localization, we conducted an analysis of the distribution of the studied CpGs across the genome (Figure 6). The data were obtained from Infinium array annotation files, where each CpG site was categorized based on its proximity to gene structures and CpG islands (Table S3).

In relation to genes, the following regions are distinguished: the region located upstream of the transcription start site (TSS) within a distance of 200–1500 bp (TSS1500), the region upstream of the TSS within a distance of 0–200 bp (TSS200), the 5′-untranslated region (5′UTR), the 1st Exon, body, and the 3′-untranslated region (3′UTR) [50]. It is important to note that, due to gene overlap, a single CpG site may be assigned to multiple regions. CpGs that are not annotated for any of these regions are considered intergenic. Based on the dinucleotide-CpG island relationship, several regions are identified: the northern and southern shores, which are located approximately 2 kb above and below the CpG island; the northern and southern shelves, which are sequences immediately adjacent to the shores and extend up to 2 kb in length; and CpG islands and the open sea, which are DNA methylation sites located outside of CpG islands.

When studying the genomic localization of CpGs in relation to the gene structure, it was found that hypermethylated CpGs are located mainly in the promoter region (TSS200), while hypomethylated ones are located in the gene body (Figure 6A). It was also found that the majority of CpGs subject to age-related hypermethylation are located in the region of CpG islands, while the hypomethylation of CpGs occurs predominantly in regions that do not contain CpG islands (Figure 6B).

## 4. Discussion

Minimized epigenetic clocks based on iPlex MassARRAY technology have been developed. This clock constructed employing the TabNet model allows us to estimate epigenetic age based on the DNA methylation levels of eight CpGs (cg07547549, cg08262002, cg01620164, cg11649376, cg16008966, cg06639320, cg14556683, and cg22454769). While our clocks may exhibit a slightly lower accuracy compared to other epigenetic clocks (best MAE = 5.99), they offer several advantages such as a simplified sample preparation process, streamlined result analysis, and reduced financial costs (for example, compared to genome-wide methylation analysis). In addition to a cost-effective approach, the minimized clock based on iPlex MassARRAY avoids data redundancy and provides researchers with flexibility, as tests are not pre-loaded on the chip by the manufacturer [26]. It allows researchers to target specific CpGs of interest.

In this study, we conducted a comparison of methylation values obtained from iPlex MassARRAY and Illumina EPIC array technologies for the same subjects, which is a novel investigation in the field. We observed significant differences between the two technologies, with maximum correlation coefficients ranging from 0.5 to 0.6. These discrepancies can be attributed to the peculiarities of each technology, as the iPlex MassARRAY and Illumina EPIC array rely on different principles for methylation assessment. Notably, the largest differences were observed for CpGs cg01620164 and cg14556683, as the iPlex MassARRAY data exhibited substantial underestimation compared to the results obtained from the Illumina 450K and EPIC BeadChips. Interestingly, even within the same technology, slight variations can lead to different outcomes. For instance, it was shown that Illumina 450K and EPIC BeadChips technologies, in general, have high correlations between results (r > 0.99), but, for many individual CpGs, the values of correlation coefficients were low (r = 0.24), and even negative [51]. Although minimized epigenetic clocks based on MassARRAY (EpiTYPER) technology have been utilized in previous studies [22,23,52], we did not find any information on the development of epigenetic clocks specifically using the iPlex MassARRAY technology. Both technologies are based on mass spectrometry and share similarities in sample preparation processes; however, EpiTYPER technology does not enable the simultaneous assessment of methylation in multiple CpGs across different genes. But, at the same time, the iPlex MassARRAY technology was successfully used in previous studies to determine somatic mutations in cancer [28,53,54], and to search for the genetic causes of non-syndromic hearing loss [26], phenylketonuria [55], rheumatoid arthritis [56], cognitive impairment [54], ischemic stroke [57], and alopecia areata [58]. On the other hand, iPlex MassARRAY technology has also been used to assess the degree of DNA methylation [59,60]. Thus, this technology can be a reliable tool for determining the level of DNA methylation as a single-nucleotide polymorphism. The clock we built using data obtained using iPlex MassARRAY technology may be useful not only for predicting epigenetic age, but also for assessing the influence of various factors on the rate of aging.

We also analyzed the distribution of the studied CpGs in relation to the gene structure and CpG island. Our findings revealed that the majority of hypermethylated sites were located in the promoter region of the gene (TSS200) and were part of CpG islands. Conversely, the hypomethylation sites were predominantly located in the gene body and were not associated with CpG islands. A similar distribution pattern of CpGs relative to CpG islands is also observed in other literature data, while age-related hypermethylation was recorded mainly at the transcription start point and the first exon of genes, and hypomethylation was registered in the gene body and regions outside of genes [43]. DNA hypermethylation was observed in 95% of age-associated CpGs within CpG islands, whereas hypomethylation was predominant outside of CpG islands, including enhancers and regions bordering transcription start sites [61].

It has been found that CpG islands are associated with 60–70% of gene promoters and are typically unmethylated [62]. It is noteworthy that the pypermethylation of CpGs within islands often accompanies cancer [63], suggesting a relationship between aging and oncogenesis. The significance of DNA methylation in intergenic and intragenic regions has been increasingly recognized due to its impact on gene expression [64]. It has been observed that methylation within the gene body is positively correlated with gene expression and is not indicative of transcriptional repression. Interestingly, genes with moderate expression levels exhibit the highest levels of intragenic methylation, while genes with both low and high expression levels tend to have lower levels of methylation [65].

CpG cg08262002, which is associated with the *LDB2* gene, has been identified as one of the top ten age-associated CpGs [66]. In addition, this CpG was included in the list of CpGs associated not only with aging but also with the development of rheumatoid arthritis [67]. A high correlation with age for cg08262002 (r = −0.69) and an even stronger correlation (r = −0.72) for the neighboring CpG site (*LDB2*_3) have been shown [68]. The *LDB2* gene encodes the LIM domain-binding protein 2, which plays a role in transcriptional regulation.

CpG cg11649376 has been identified as a leading CpG site associated with inflammation and obesity [69]. The *ACSS3* gene containing this CpG site encodes a protein that catalyzes fatty acid metabolism and degrades ketone bodies, resulting in energy production [70]. CpG cg11649376, along with CpGs cg08262002, cg22454769, cg06639320, and cg08262002, has been selected for age prediction not only in healthy individuals, but also in patients with rheumatoid arthritis [67].

CpG cg16008966 has been included in the top 53 CpGs showing intra-individual longitudinal changes [71]. It has demonstrated a significant correlation with age, even when using a model that considers age-related changes in cellular composition [72]. CpGs cg06639320 and cg22454769 are associated with the *FHL2* gene, which encodes a transcription factor involved in the regulation of cell differentiation. The hypermethylation of CpGs in *FHL2*, along with *ELOVL2*, is considered one of the most significant epigenetic changes associated with aging [9]. Numerous studies have identified CpGs cg06639320 and cg22454769 as highly significant in the aging process [73,74,75]. The correlation between the methylation level at these sites and age has been observed not only in whole blood, but also in other tissues [76].

The *SLC12A5* gene, which contains CpG cg07547549, encodes a neuron-specific membrane protein called K^+^/Cl^−^ cotransporter (KCC2). This protein plays a crucial role in maintaining the correct inhibitory function of the neurotransmitters γ-aminobutyric acid and glycine, as well as regulating the intracellular concentration of Cl^−^ in neurons [77]. It is noteworthy that CpG cg07547549 has been included in minimized epigenetic clocks not only for whole blood [78], but also for saliva [79], and for hair [80].

CpG cg01620164, associated with the *FIGN* gene, has been shown to have a relationship not only with age, but also with sex [81]. The methylation level of CpG cg01620164 showed the highest negative correlation with age (r = −0.64) [14]. However, in our study, no statistically significant correlation with age was observed. The product of the *FIGN* gene is involved in essential cellular processes such as mitosis, meiosis, DNA synthesis, and cell migration, and its overexpression may contribute to tumor progression [82].

The *EPHX3* gene, which contains CpG cg14556683, encodes epoxide hydrolase 3, an enzyme involved in the hydrolysis of fatty acid epoxides. Notably, *EPHX3* hypermethylation has been associated with the development of certain cancers. *EPHX3* methylation was proposed as a prognostic marker for head and neck cancer [83]. Additionally, age-dependent hypermethylation of CpG cg14556683 has been observed in various brain regions [84].

It is interesting to note that individual CpGs may have a relatively weak correlation with chronological age, but the overall accuracy of an epigenetic clock can be quite high due to the large number of CpG sites. For instance, Hannum and Horvath clocks have achieved correlation coefficients with age exceeding 0.9 and mean errors of less than five years [12,13].

We acknowledge that our study presents some limitations. Firstly, the sample size was relatively small, and it did not include individuals from extreme age ranges. To further validate our minimized clocks, it would be beneficial to test them on accelerated and delayed aging models, such as individuals with Down syndrome, centenarians, and their descendants [22]. Secondly, for some CpGs, we obtained significantly different methylation values for the same subjects using iPlex MassARRAY and EPIC BeadChips. This highlights the need for optimization and standardization of methods. Moreover, it is important to consider the technology-specific differences and exclude region-specific CpGs when constructing an epigenetic clock to ensure its applicability across diverse populations. In future studies, we plan to investigate region-specific CpGs by comparing methylation profiles of individual CpGs in individuals from different geographical regions, such as residents of the central part of Russia and the Far North.

In conclusion, we tested the possibility of creating a minimized epigenetic clock based on the iPlex MassARRAY technology. We have demonstrated that the TabNet architecture is the best model for clock building because it produces minimal age error compared to other machine-learning models. Thus, the minimized epigenetic clock based on the iPlex MassARRAY platform is a promising predictor of age, which requires further research and improvements.

We hope that our study can be useful for the development of patient-accessible epigenetic clocks. Developing a minimized epigenetic clock is an important challenge because such clocks can provide a sufficient accuracy of age estimation while also reducing research costs.

## Figures and Tables

**Table 2:** Chronological age regression according to iPlex MassARRAY data.

GSE152026 MAE	GSE55763 MAE	GSE40279 MAE	GSE87571 MAE	UNN EPIC MAE	UNN MassARRAY Validation Best MAE	UNN MassARRAY Validation (MAE) ± STD	Model	Type
14.98	8.67	8.71	11.97	12.00	9.67	11.23 ± 1.37	Elastic Net	Linear
10.93	6.83	10.13	8.12	10.95	8.20	11.61 ± 3.41	XGBoost	GBDT
9.93	14.00	9.36	11.75	10.13	6.91	11.94 ± 3.35	LightGBM	
10.46	5.07	8.16	6.59	8.50	6.07	9.37 ± 2.94	CatBoost	
10.65	9.48	10.72	9.41	8.31	7.98	10.62 ± 1.54	MLP	DNN
8.34	7.67	7.13	6.83	8.08	5.99	8.67 ± 2.65	TabNet	
7.73	7.28	6.30	7.46	6.22	6.12	9.25 ± 3.28	FT-Transformer	

**Table 3:** MAE values for different epigenetic clock models.

GSE152026	GSE55763	GSE40279	GSE87571	UNN EPIC	Clock
8.34	7.67	7.13	6.83	8.08	MassARRAY Age
5.05	7.02	4.73	5.77	13.45	DNAmAgeHannum
8.85	6.20	5.34	4.70	6.41	DNAmAge
6.01	4.97	7.95	5.03	10.94	DNAmPhenoAge
4.73	5.25	8.89	7.57	13.17	DNAmGrimAge
11.28	5.50	6.35	6.19	8.04	PCHorvath1
8.58	4.82	6.77	7.52	10.48	PCHorvath2
12.57	8.76	5.65	10.33	7.65	PCHannum
5.05	4.29	6.82	5.22	4.93	PCPhenoAge
15.74	12.87	10.59	12.19	8.60	PCGrimAge

## Data Availability

The methylation data of the selected eight CpGs for the UNN MassARRAY dataset are presented in Table S4. The genome-wide methylation data of the used open datasets are available in the Gene Expression Omnibus repository (GSE40279, GSE87571, GSE55763 and GSE152026). The genome-wide methylation data for subjects from the Nizhny Novgorod laboratory (UNN EPIC dataset) are available upon individual request to the email address: spring_dusk@mail.ru.

## Supplementary Materials

The following supporting information can be downloaded at: https://www.mdpi.com/article/10.3390/genes15040425/s1, Figure S1 Work efficiency of different epigenetic clocks on considered datasets (GSE87571, GSE40279, GSE55763, and GSE152026). Table S1. MassArray design details. Table S2. Description of datasets. Table S3. Localization of the studied CpGs. Table S4. Methylation level (%) of studied CpGs in UNN MassARRAY dataset.

## Abbreviations

bp—base pair, CatBoost—categorical boosting, CpGs—CpG sites, ddNTP—dideoxynucleotide triphosphate, DNN—deep neural network, dNTP—deoxynucleotide triphosphate, FT-Transformer—feature tokenizer and transformer, GBDT—gradient-boosted decision tree, I—inosine, LightGBM—light gradient-boosting machine, MAE—mean absolute error, MALDI-TOF MS—matrix-assisted laser desorption/ionization time-of-flight mass spectrometry, 5mC—5-methylcytosine, MLP—multilayer perceptron, MS-qPCR—quantitative PCR method for assessing methylation, SAP—shrimp alkaline phosphatase, SNP—single-nucleotide polymorphism, TSS—transcription start site, UTR—untranslated region, UV—ultraviolet, XGBoost—extreme gradient boosting.
