# Supplementary material for: Building Minimized Epigenetic Clock by iPlex MassARRAY Platform
Source: Genes (Basel). 2024 Mar 28;15(4):425. doi: 10.3390/genes15040425 (PMC11049545; doi:10.3390/genes15040425)
Supplement: Supplementary file 1 [file genes-15-00425-s001.zip › genes-2911602-supplementary.pdf]

.Supplementary Material

Supplementary data to this article can be found online.

GSE87571

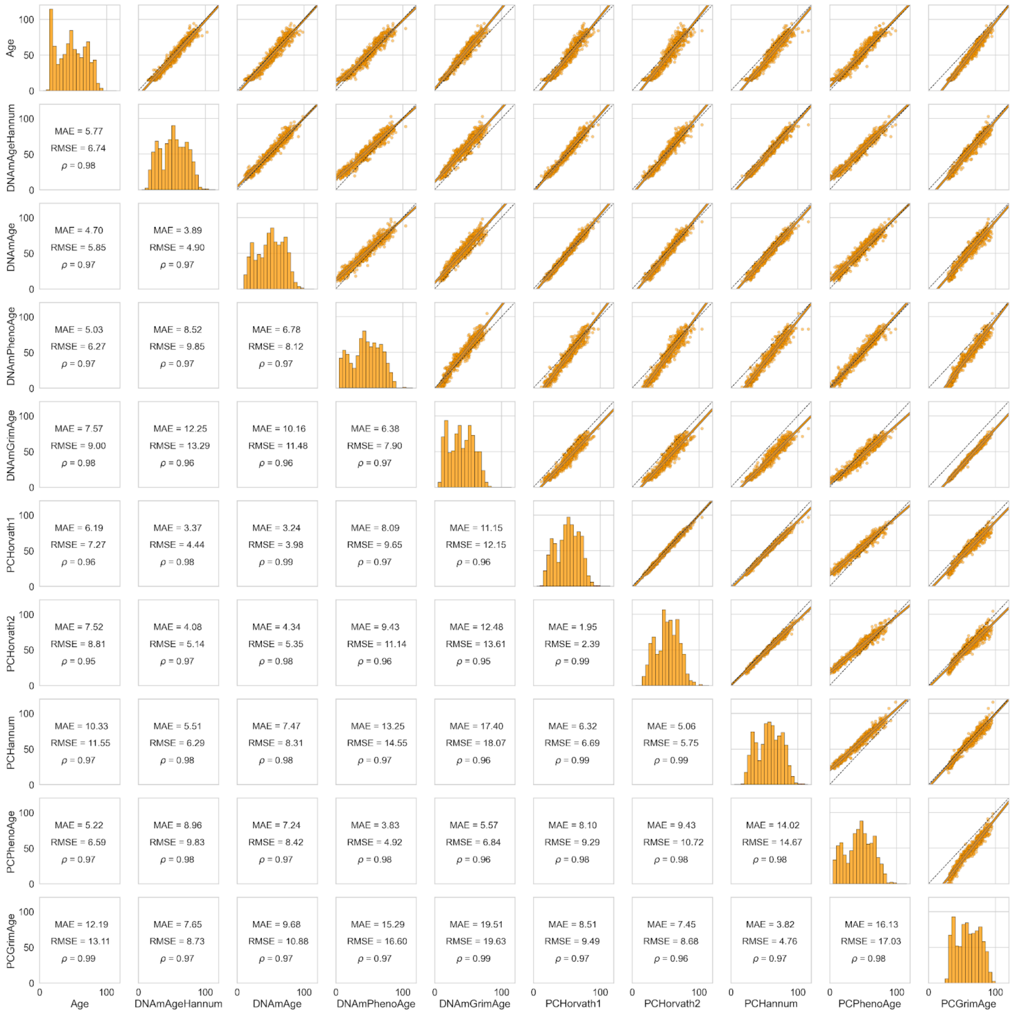

GSE40279

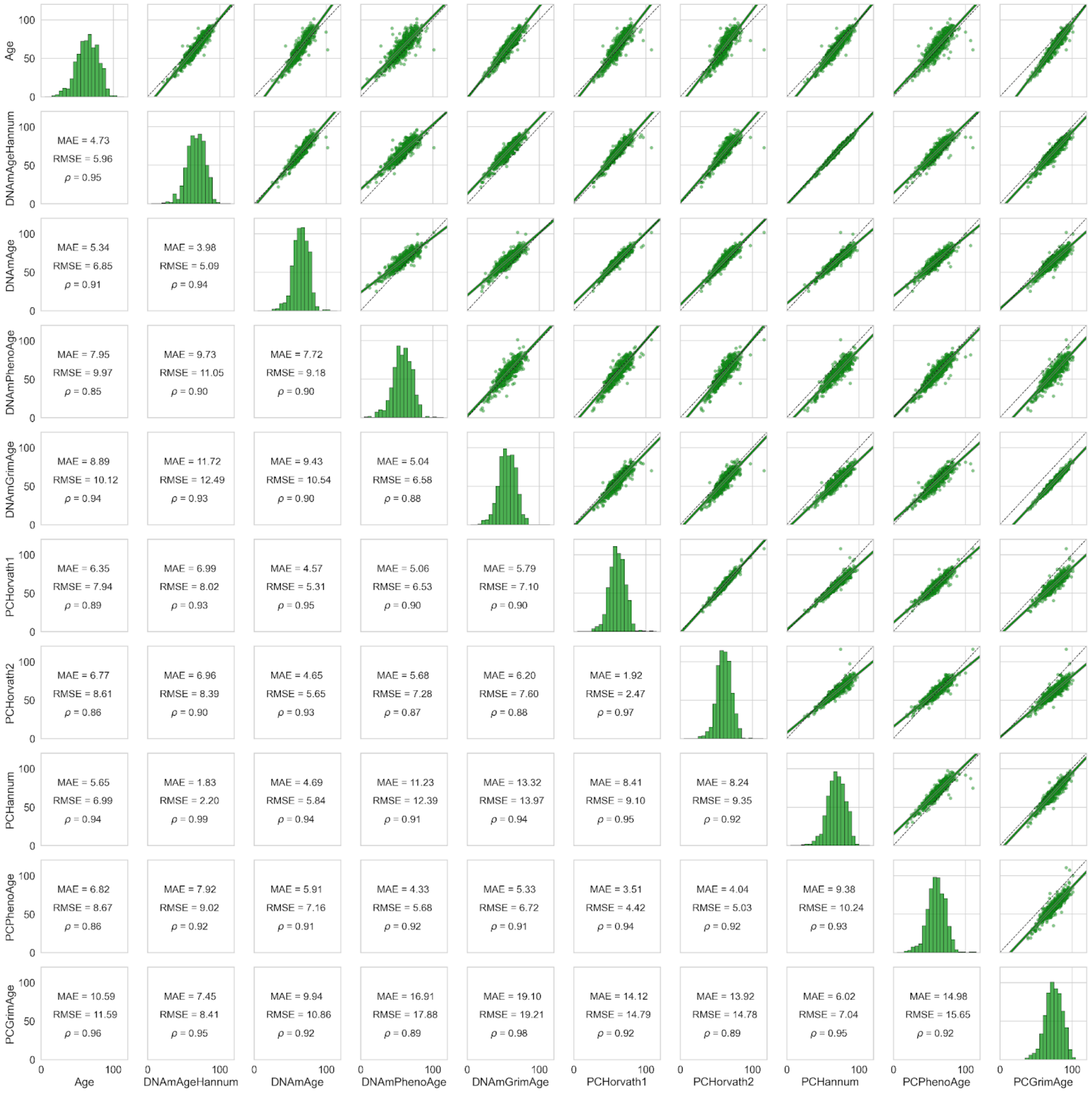

Age
DNAmAgeHannum
DNAmAge
DNAmPhenoAge
DNAmGrimAge
PCHorvath1
PCHorvath2
PCHannum
PCPhenoAge
PCGrimAge
0
50
100
MAE = 4.73 RMSE = 5.96 ρ = 0.95
MAE = 5.34 RMSE = 6.85 ρ = 0.91
MAE = 3.98 RMSE = 5.09 ρ = 0.94
MAE = 7.95 RMSE = 9.97 ρ = 0.85
MAE = 9.73 RMSE = 11.05 ρ = 0.90
MAE = 7.72 RMSE = 9.18 ρ = 0.90
MAE = 8.89 RMSE = 10.12 ρ = 0.94
MAE = 11.72 RMSE = 12.49 ρ = 0.93
MAE = 9.43 RMSE = 10.54 ρ = 0.90
MAE = 5.04 RMSE = 6.58 ρ = 0.88
MAE = 6.35 RMSE = 7.94 ρ = 0.89
MAE = 6.99 RMSE = 8.02 ρ = 0.93
MAE = 4.57 RMSE = 5.31 ρ = 0.95
MAE = 5.06 RMSE = 6.53 ρ = 0.90
MAE = 5.79 RMSE = 7.10 ρ = 0.90
MAE = 6.77 RMSE = 8.61 ρ = 0.86
MAE = 6.96 RMSE = 8.39 ρ = 0.90
MAE = 4.65 RMSE = 5.65 ρ = 0.93
MAE = 5.68 RMSE = 7.28 ρ = 0.87
MAE = 6.20 RMSE = 7.60 ρ = 0.88
MAE = 1.92 RMSE = 2.47 ρ = 0.97
MAE = 5.65 RMSE = 6.99 ρ = 0.94
MAE = 1.83 RMSE = 2.20 ρ = 0.99
MAE = 4.69 RMSE = 5.84 ρ = 0.94
MAE = 11.23 RMSE = 12.39 ρ = 0.91
MAE = 13.32 RMSE = 13.97 ρ = 0.94
MAE = 8.41 RMSE = 9.10 ρ = 0.95
MAE = 8.24 RMSE = 9.35 ρ = 0.92
MAE = 6.82 RMSE = 8.67 ρ = 0.86
MAE = 7.92 RMSE = 9.02 ρ = 0.92
MAE = 5.91 RMSE = 7.16 ρ = 0.91
MAE = 4.33 RMSE = 5.68 ρ = 0.92
MAE = 5.33 RMSE = 6.72 ρ = 0.91
MAE = 3.51 RMSE = 4.42 ρ = 0.94
MAE = 4.04 RMSE = 5.03 ρ = 0.92
MAE = 9.38 RMSE = 10.24 ρ = 0.93
MAE = 10.59 RMSE = 11.59 ρ = 0.96
MAE = 7.45 RMSE = 8.41 ρ = 0.95
MAE = 9.94 RMSE = 10.86 ρ = 0.92
MAE = 16.91 RMSE = 17.88 ρ = 0.89
MAE = 19.10 RMSE = 19.21 ρ = 0.98
MAE = 14.12 RMSE = 14.79 ρ = 0.92
MAE = 13.92 RMSE = 14.78 ρ = 0.89
MAE = 6.02 RMSE = 7.04 ρ = 0.95
MAE = 14.98 RMSE = 15.65 ρ = 0.92

GSE55763

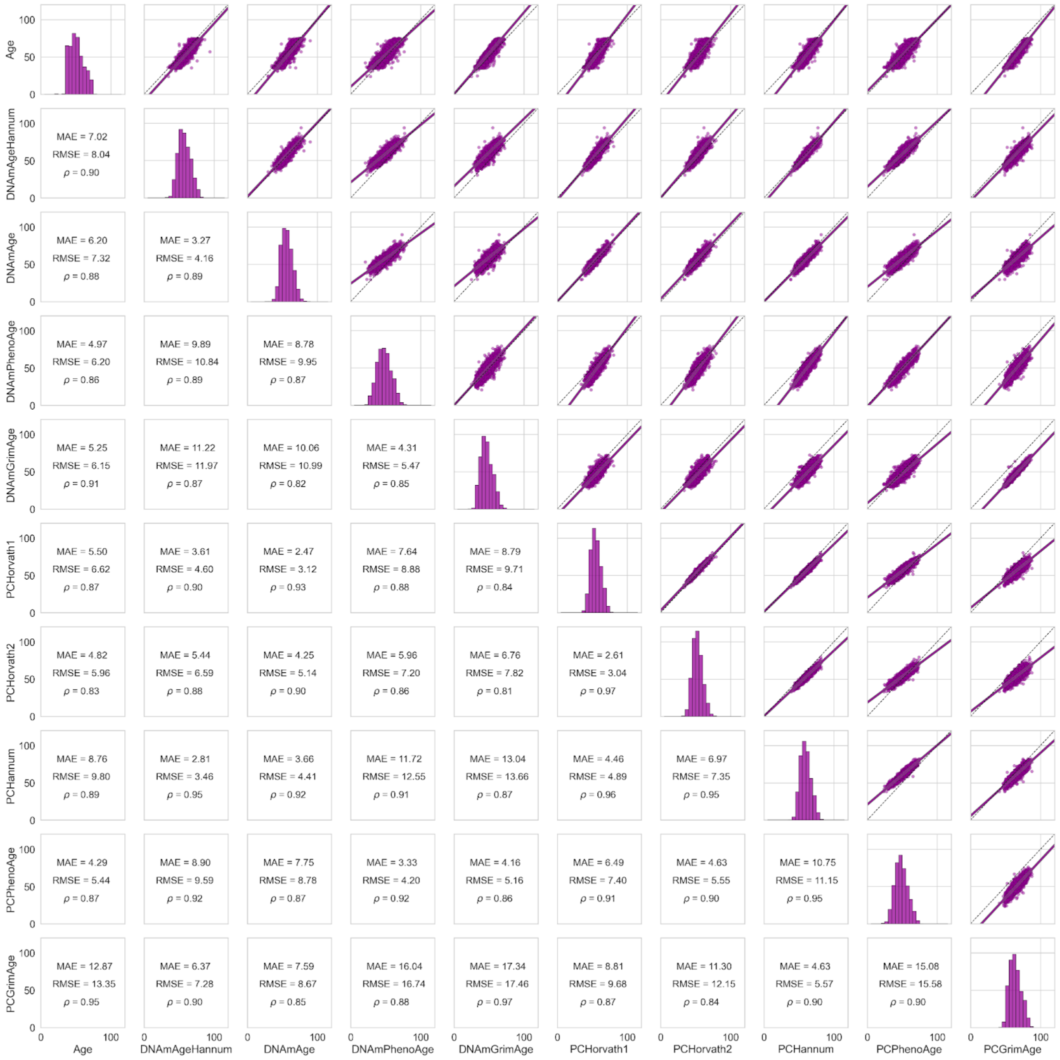
Age
DNAmAgeHannum
DNAmAge
DNAmPhenoAge
DNAmGrimAge
PCHorvath1
PCHorvath2
PCHannum
PCPhenoAge
PCGrimAge
0
50
100
MAE = 7.02 RMSE = 8.04 ρ = 0.90
MAE = 6.20 RMSE = 7.32 ρ = 0.88
MAE = 3.27 RMSE = 4.16 ρ = 0.89
MAE = 4.97 RMSE = 6.20 ρ = 0.86
MAE = 9.89 RMSE = 10.84 ρ = 0.89
MAE = 8.78 RMSE = 9.95 ρ = 0.87
MAE = 5.25 RMSE = 6.15 ρ = 0.91
MAE = 11.22 RMSE = 11.97 ρ = 0.87
MAE = 10.06 RMSE = 10.99 ρ = 0.82
MAE = 4.31 RMSE = 5.47 ρ = 0.85
MAE = 5.50 RMSE = 6.62 ρ = 0.87
MAE = 3.61 RMSE = 4.60 ρ = 0.90
MAE = 2.47 RMSE = 3.12 ρ = 0.93
MAE = 7.64 RMSE = 8.88 ρ = 0.88
MAE = 8.79 RMSE = 9.71 ρ = 0.84
MAE = 4.82 RMSE = 5.96 ρ = 0.83
MAE = 5.44 RMSE = 6.59 ρ = 0.88
MAE = 4.25 RMSE = 5.14 ρ = 0.90
MAE = 5.96 RMSE = 7.20 ρ = 0.86
MAE = 6.76 RMSE = 7.82 ρ = 0.81
MAE = 2.61 RMSE = 3.04 ρ = 0.97
MAE = 8.76 RMSE = 9.80 ρ = 0.89
MAE = 2.81 RMSE = 3.46 ρ = 0.95
MAE = 3.66 RMSE = 4.41 ρ = 0.92
MAE = 11.72 RMSE = 12.55 ρ = 0.91
MAE = 13.04 RMSE = 13.66 ρ = 0.87
MAE = 4.46 RMSE = 4.89 ρ = 0.96
MAE = 6.97 RMSE = 7.35 ρ = 0.95
MAE = 4.29 RMSE = 5.44 ρ = 0.87
MAE = 8.90 RMSE = 9.59 ρ = 0.92
MAE = 7.75 RMSE = 8.78 ρ = 0.87
MAE = 3.33 RMSE = 4.20 ρ = 0.92
MAE = 4.16 RMSE = 5.16 ρ = 0.86
MAE = 6.49 RMSE = 7.40 ρ = 0.91
MAE = 4.63 RMSE = 5.55 ρ = 0.90
MAE = 10.75 RMSE = 11.15 ρ = 0.95
MAE = 12.87 RMSE = 13.35 ρ = 0.95
MAE = 6.37 RMSE = 7.28 ρ = 0.90
MAE = 7.59 RMSE = 8.67 ρ = 0.85
MAE = 16.04 RMSE = 16.74 ρ = 0.88
MAE = 17.34 RMSE = 17.46 ρ = 0.97
MAE = 8.81 RMSE = 9.68 ρ = 0.87
MAE = 11.30 RMSE = 12.15 ρ = 0.84
MAE = 4.63 RMSE = 5.57 ρ = 0.90
MAE = 15.08 RMSE = 15.58 ρ = 0.90

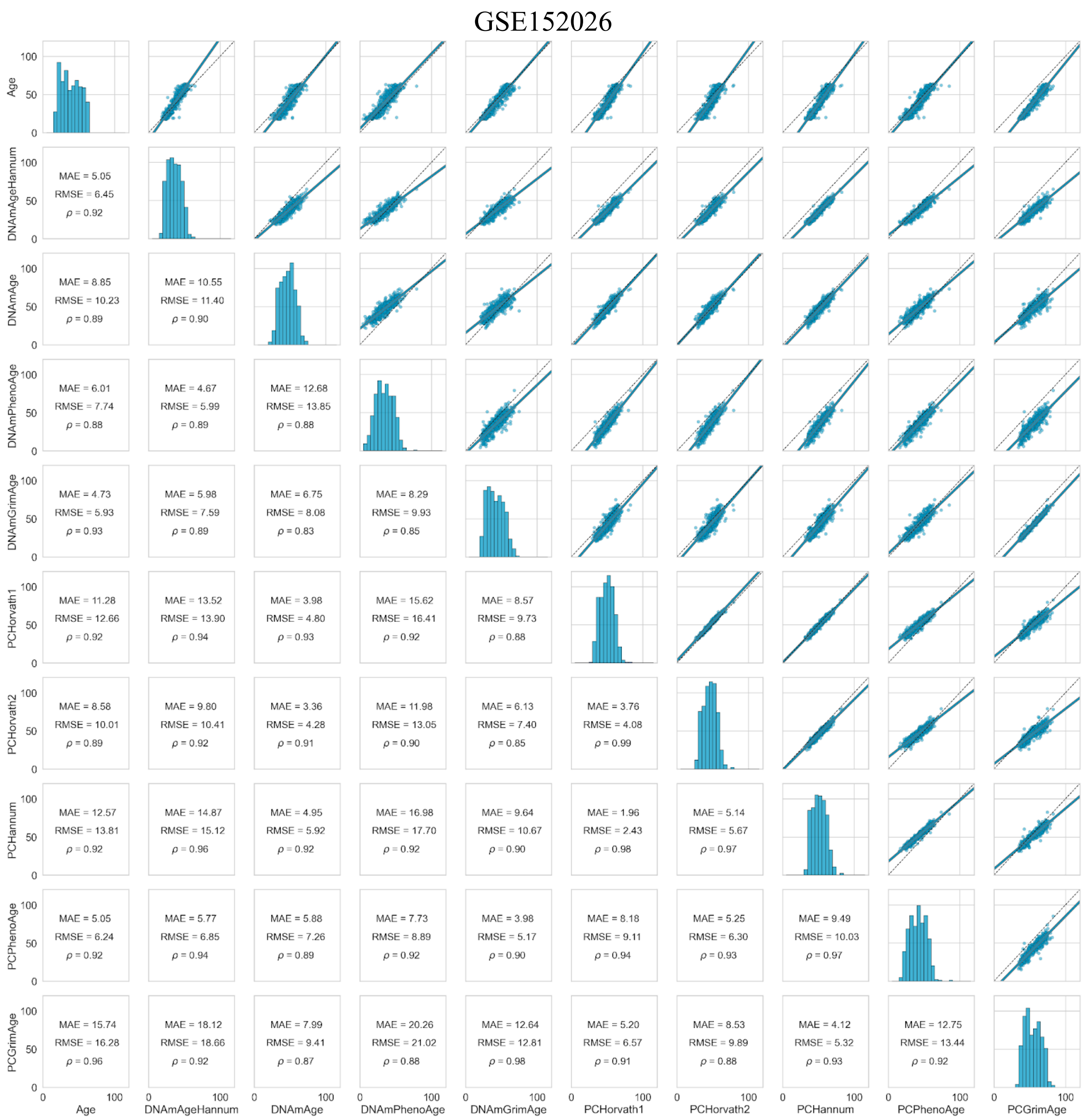

Figure S1. Work efficiency of different epigenetic clocks on considered datasets (GSE87571, GSE40279, GSE55763, and GSE152026). The left side of these figures indicates four types of epigenetic clock from Horvath's calculator—DNAmAgeHannum, DNAmAge, DNAmPhenoAge, and DNAmGrimAge—and its five PC variations. The first column shows error values relative to chronological age (MAE), root-mean-squared error (RMSE), and correlation between different epigenetic clock models.

Table S1. MassArray design details.

Extension Primers (5′-3′)	Tm, °C	Amplicon Length (bp)	Forward and Reverse Primers (5′-3′)	CpG_ID
TGG GGT GTT TAA GGT<br>GTT GGA ATG	58.7<br>63	189	ACG TTG GAT GGT TGT AGT TTG GAG TTT AG<br>ACG TTG GAT GAT CTT CTA TCC CCT TCC CAC	cg07547549
GTG GTT TTT GTT TTT<br>TTA GAA	61.6<br>60.4	131	ACG TTG GAT GGG TAT TGA GTG AGG TAT AGG<br>ACG TTG GAT GCC ATT CAC CAT TCA TAC ATT C	cg08262002
GAT GAA GAG AGG AAT<br>TAT AAG ATT	63<br>60.3	136	ACG TTG GAT GGT TAG GGG TTT GTA GTG TGG<br>ACG TTG GAT GTC ACC AAA ATC CAA CCA AAC	cg01620164
GTT AGG TTT GTA GTG<br>GA	60.3<br>60.3	118	ACG TTG GAT GGT TTA GTA ATG AGA TGA GGG<br>ACG TTG GAT GAC TCT AAC CAA TTC CAT TCC	cg11649376
CCC CCG TGT ATA TGT<br>GTG TAT GTG	61.6<br>60.4	124	ACG TTG GAT GTG AGT GTG GAG GAA TGA ATG<br>ACG TTG GAT GCT TTC ATT CTT ACT CTA CCT C	cg16008966
GTT TTG GGA GTA TAG<br>TAG TTA T	56.2<br>61.5	153	ACG TTG GAT GGT TTG TTA GGG TTT TTT TTT<br>ACG TTG GAT GTA AAC AAA CCC CTA CCA CC	cg06639320
GTA GTA GTT TTA GIG<br>ATA GG	56.2<br>61.7	158	ACG TTG GAT GTA TTA GGT TTT ATA TGA AGG<br>ACG TTG GAT GCT ACT ACA AAT ACC CCT AAC C	cg14556683
TTT CGG IGT GGG TTT T	56.2<br>61.5	153	ACG TTG GAT GGT TTG TTA GGG TTT TTT TTT<br>ACG TTG GAT GTA AAC AAA CCC CTA CCA CC	cg22454769

Table S2. Description of datasets.

Number of Probes After Preprocessing	Number of Subjects	Methylation Data Details	Dataset	Illumina
411,974	729	Raw .idat files, functional normalization	GSE87571	
412,039	656	No raw .idat files, no normalization, adjusting for internal controls by the Illumina's Genome Studio software.	GSE40279	450K
412,340	2639	No raw .idat files, quantile normalization	GSE55763	
739,168	131	Raw .idat files, functional normalization	UNN	EPIC
743,182	519 (Controls)	No raw .idat files, normalization: dasen from wateRmelon	GSE152026	

Table S3. Localization of the studied CpGs.

Relation_to_UCSC_CpG_Island	UCSC_RefGene_Group	UCSC_RefGene_Accession	UCSC_RefGene_Name	Position	CpG_ID
Island	Body (intron); Body (intron)	NM_020708; NM_001134771	*SLC12A5*; *SLC12A5*	chr20:44658226	cg07547549
Open_sea	Body (intron); Body (intron)	NM_001290; NM_001130834	*LDB2*; *LDB2*	chr4:16575323	cg08262002
N_Shelf	Body (intron)	NM_018086	*FIGN*	chr2:164590273	cg01620164
S_Shore	Body (intron)	NM_024560	*ACSS3*	chr12:81473234	cg11649376
Open_sea	-	-	-	chr1:114761794	cg16008966
Island	TSS200; TSS200; 5′UTR; TSS200	NM_001039492; NM_001450; NM_201557; NM_201555	*FHL2*; *FHL2*; *FHL2*; *FHL2*	chr2:106015740	cg06639320
Island	1stExon; Body (exon)	NM_024794; NM_001142886	*EPHX3*; *EPHX3*	chr19:15342983	cg14556683
Island	TSS200; TSS200; 5′UTR; TSS200	NM_001039492; NM_001450; NM_201557; NM_201555	*FHL2*; *FHL2*; *FHL2*; *FHL2*	chr2:106015768	cg22454769

Table S4. Methylation level (%) of studied CpGs in UNN MassARRAY dataset.

cg22454769	cg14556683	cg06639320	cg16008966	cg11649376	cg01620164	cg08262002	cg07547549	Age	Sex	Sample
24.69	9.12	30.15	57.88	53.36	23.08	44.22	31.19	24.72	m	I21
35.36	5.49	28.72	70.40	61.32	25.49	59.74	40.77	24.73	m	I42
47.57	5.52	45.43	55.34	62.27	24.01	52.15	28.98	24.99	f	I31

33.17	2.09	33.69	64.54	55.21	25.83	51.43	28.36	25.35	f	I55
45.76	6.35	41.98	60.86	64.00	25.13	62.45	35.31	26.01	m	I33
39.41	4.17	35.96	53.42	43.30	24.73	46.66	41.32	29.25	m	I11
39.78	8.88	34.87	58.43	59.47	25.68	57.98	26.45	30.9	f	I28
35.66	1.97	38.82	58.67	54.24	22.98	46.76	57.30	32.24	m	I34
36.80	0	39.47	55.77	51.03	28.90	41.22	33.69	32.78	f	I2
32.38	10.57	41.99	56.85	51.61	23.37	47.02	47.49	32.85	m	I14
42.29	1.11	16.23	64.42	64.10	39.85	72.12	19.93	33.99	f	I27
44.86	5.32	31.95	67.61	59.84	47.70	63.68	30.77	36.84	f	I19
26.74	0	40.29	56.72	54.81	22.74	44.31	48.97	38.54	f	I45
40.57	3.80	25.05	65.29	68.96	15.88	81.16	24.06	38.87	m	I1
41.05	2.01	41.09	61.64	50.76	24.01	46.74	49.51	41.54	f	I29
39.69	1.31	48.02	61.66	60.12	24.94	48.89	42.82	41.79	m	I4
41.33	1.34	39.20	60.03	59.19	47.29	59.65	23.64	42.07	m	I7
50.70	10.44	30.29	57.05	56.84	6.16	65.32	25.31	42.45	m	I32
42.04	5.88	39.87	63.96	54.78	25.79	49.68	30.08	42.89	m	I62
52.66	1.31	50.94	61.94	57.65	26.53	54.80	35.43	47.6	f	I20
42.20	6.86	48.37	55.83	55.74	25.30	44.96	36.42	48.3	f	I48
39.92	3.12	32.20	61.30	52.08	25.85	57.58	9.13	49.74	m	I47
49.83	0	41.63	69.47	56.82	25.95	80.11	32.12	50.18	f	I63
47.80	0	39.67	62.92	50.56	20.87	45.34	42.09	50.26	f	I54
37.46	4.96	64.28	59.23	54.69	31.97	45.02	57.14	53.53	f	I5
57.52	0	17.35	14.68	49.23	11.87	72.70	40.44	53.83	m	I6
30.12	2.59	54.96	58.03	52.09	27.02	55.08	45.98	55.49	m	I38
56.36	6.50	56.10	51.57	43.99	18.90	37.16	30.28	55.52	f	I16
34.54	30.54	55.11	55.73	61.79	34.86	34.44	53.12	55.58	f	I39
46.51	6.06	49.53	64.71	54.10	26.90	51.90	44.83	55.68	f	I44
49.97	7.75	47.98	55.79	49.17	25.41	43.25	59.70	58.9	m	I17
52.78	15.41	31.51	63.24	50.70	26.69	59.41	21.02	60.51	f	I61
51.49	8.64	48.65	53.75	52.56	24.21	51.50	47.18	61.64	f	I53
50.91	10.46	31.99	68.50	55.27	35.95	71.39	30.10	64.03	f	I56
55.08	0	49.47	69.11	59.54	33.99	62.83	33.01	64.49	f	I51
53.76	6.02	53.98	56.10	53.50	26.60	53.02	37.13	66.85	m	I69
51.69	10.62	57.70	62.89	59.54	26.74	52.18	39.07	67.12	f	I52
57.81	0	51.81	86.97	52.29	13.65	67.67	38.19	68.09	m	I67
47.80	4.96	51.39	58.22	48.04	26.16	46.38	35.80	69.52	f	I73
55.55	6.51	43.15	51.66	47.37	31.44	60.02	27.25	69.99	f	I59
41.42	0.49	36.04	34.32	35.20	23.98	35.28	43.04	70.68	m	I25
39.13	7.78	41.02	55.81	53.45	45.46	50.66	31.54	72.26	f	I71
57.95	3.13	67.38	60.24	53.28	24.48	44.56	40.98	74.35	f	I100
59.45	5.02	55.20	41.77	54.21	24.21	40.05	61.24	77.11	m	I76
42.41	0	41.32	35.14	32.67	22.21	36.59	29.01	78.08	f	I43
71.60	10.16	69.08	41.83	56.75	17.05	43.54	40.66	79.71	m	I94
47.39	11.27	58.30	46.95	46.61	27.72	35.18	32.38	82.15	f	I96
49.57	10.79	57.13	57.25	53.13	24.46	43.96	42.13	82.87	m	I92
54.91	31.05	62.44	50.13	46.45	38.56	30.80	42.64	83.53	f	I93
48.05	12.55	60.07	47.03	47.43	29.48	32.69	40.31	83.64	f	I95
